# Immune defense in *Drosophila melanogaster* depends on diet, sex, and mating status

**DOI:** 10.1371/journal.pone.0268415

**Published:** 2023-04-13

**Authors:** Kshama Ekanath Rai, Han Yin, Arnie Lynn C. Bengo, Madison Cheek, Robert Courville, Elnaz Bagheri, Reza Ramezan, Sam Behseta, Parvin Shahrestani

**Affiliations:** 1 Department of Biological Sciences, California State University, Fullerton, Fullerton, California, United States of America; 2 Department of Mathematics, California State University, Fullerton, Fullerton, California, United States of America; 3 Department of Statistic and Actuarial Science, University of Waterloo, Ontario, Canada; University of Saskatchewan College of Agriculture and Bioresources, CANADA

## Abstract

Immune defense is a complex trait that affects and is affected by many other host factors, including sex, mating, and dietary environment. We used the agriculturally relevant fungal emtomopathogen, *Beauveria bassiana*, and the model host organism *Drosophila melanogaster* to examine how the impacts of sex, mating, and dietary environment on immunity are interrelated. We showed that the direction of sexual dimorphism in immune defense depends on mating status and mating frequency. We also showed that post-infection dimorphism in immune defense changes over time and is affected by dietary condition both before and after infection. Supplementing the diet with protein-rich yeast improved post-infection survival but more so when supplementation was done after infection instead of before. The multi-directional impacts among immune defense, sex, mating, and diet are clearly complex, and while our study shines light on some of these relationships, further study is warranted. Such studies have potential downstream applications in agriculture and medicine.

## Introduction

Immune defense is a complex trait that interacts with many other aspects of an organism’s biology, including sex, mating, and dietary condition, all of which can affect resource availability and allocation, hormonal signaling, and other factors known to be involved in fighting infections. Experimentally testing the specific impacts of sex, mating, and dietary condition on immune defense calls for the use of model organisms. The laboratory fruit fly, *Drosophila melanogaster*, provides a suitable model for such studies, due to its affordability, ease of use, and homology with mammalian innate immunity [1, 2].

In *D*. *melanogaster*, sexual dimorphism in immune defense has been documented by several research laboratories [3–5], and sometimes attributed to sexual selection [6]. Yet some studies do not find sexual dimorphism in immunity, or show that the direction (which sex is more susceptible) and magnitude of sexual dimorphism in immune defense depends on many factors including host genotype [7, 8].

Like sex, mating also can affect immunity in complex ways in both females and males [9–11]. The relationship between mating and immunity is often bi-directional, in that an immune response reduces reproductive output, whereas mating and reproduction suppress immune defense [9]. Yet some studies suggest that mating improves immunity [9, 12, 13], or doesn’t affect it at all [14–16].

Both sexual dimorphism and mating-immunity trade-offs are non-axiomatic, and appear to depend on how immune defense is measured [9], what pathogen is used [13, 17], and likely on the environmental context, including diet. Research laboratories that use the *D*. *melanogaster* model use various diets even when they use the same fly genotypes [18]. These diet choices are typically made based on the history of the facility, generally without explicitly considering the biological impact of the specific diet relative to any other diet. Yet diet may have important impacts on female and male life histories, and on the balance between reducing costs associated with pathogenicity versus the costs of immunity itself [19]. Insect immune defense is energetically costly, with resistance to infection being affected by diet [20–22]. On low protein diets (low in yeast), *D*. *melanogaster* with high bacterial-resistance have low uninfected fecundity [21, 23]. But on yeast-rich diets, *D*. *melanogaster* have both high fecundity and strong immune defense [21]. Both baseline and infection-induced sex differences in nutrient preference and uptake may further complicate the sex- and mating- related impacts on immunity [24].

We previously showed that infection with various strains of *B*. *bassiana*, a fungal entomopathogen used in biological control of insects, elicits a sexually dimorphic immune response, but the magnitude of sexual dimorphism depends on the specific fungal strain, and the dimorphism can be ablated by specific genetic mutations in the host [7]. Later we showed that recombinant inbred lines from the *Drosophila* Synthetic Population Resource (DSPR) differed in whether or not they experienced sexual dimorphism in immunity; and when there was sexual dimorphism the direction and magnitude of it depended on host genotype [8]. While fungal infections continue to be understudied relative to other *D*. *melanogaster* infections [3], others too have shown the importance of diet in sex-based fungal infection outcomes [25, 26].

In this study we first replicated our previous work [7] showing the existence of sexual dimorphism in response to fungal infection. We next tested how this dimorphism is affected by mating status, comparing virgin, short-term mated, and long-term mated flies. Mating status affected post-infection survival and also affected the direction of sexual dimorphism in immune defense. The impact of prolonged mating was not explained solely by changes to reproductive output. Next, we examined the effects of diet on sexual dimorphism in immune defense. The effects of diet on post-infection survival were sex-specific and depended on whether the dietary manipulation occurred before or after pathogen exposure. Overall, our study contributes to understanding the complex interactions among sex, mating, and diet as they impact immune defense.

## Materials and methods

### Drosophila melanogaster population

We used a genetically diverse *D*. *melanogaster* population, the C3 population of Shahrestani et al. [8]. Population C3 is maintained with non-overlapping 14-day generations at population size of 2,000 flies at ~25° C, 12h light:12h dark cycles. For the past 72 generations, Population C3 was maintained at the California State University in Fullerton on a Cornmeal (C) diet (per L of deionized water: 12 g agar, 29 g yeast, 71 g cornmeal, 92 g molasses, 0.66 mL acid mix composed of 45% phosphoric acid by volume and 0.16 mL Tegosept), and prior to that it was maintained at Cornell University on a Glucose (G) diet for at least 200 generations (per liter of deionized water: 100 g glucose, 100 g Brewer’s yeast, 12 g agar, 0.84% propionic acid, and 0.08% phosphoric acid). At the start of each experiment, flies were reared in vials at densities of 60–80 eggs per vial as in our past work [8].

Two strains of the entomopathogenic fungus *Beauveria bassiana* were used, as in our past work [7]: GHA, which was obtained from Mycotech, Inc. (now Bioworks, Inc., Victor NY, lot number TGA1-96-06B), and ARSEF 12460 (Shahrestani and Vandenberg; USDA Agricultural Research Service Collection of Entomopathogenic Fungi, Ithaca, NY). Fungal spores were stored at -20° C. Prior to use, spores were allowed to warm to room temperature.

### Fungal inoculation

Fungal inoculations were done as previously described [7, 8]. Specifically, flies were briefly anesthetized with CO_2_ and then moved onto Petri dishes placed on ice to maintain anesthesia while they were sprayed either with 5mL of a control fungal-free suspension of 0.03% Silwet (PlantMedia™, a division of bioWORLD) in autoclaved DI water, or with 5mL of a fungal suspension of 0.3g of *Beauveria bassiana* spores (either ARSEF 12460 or GHA) suspended in 25 mL of 0.03% Silwet. Sprays were done using a custom-built spray tower [27]. The spray dose was double checked by placing a microscope slide cover next to the flies during inoculation, then resuspending the slide and counting the number of spores in 10uL suspensions. Each spray introduced ~10^3^ spores/mm^2^ onto the surface of anesthetized flies. While female *D*. *melanogaster* in Population C3 are about twice as large as males, our prior work has shown that sexual dimorphism in post-infection survival stays consistent across doses varied by multiple orders of magnitude [7]. After the spray, flies from each treatment were moved to separate acrylic cages (volume: 450 cm^3^), fed with a Petri dish of fly medium, and kept at ~100% humidity for 24 hours. In high humidity conditions, fungal conidia germinate, and the hyphae penetrate the insect cuticle, entering the hemocoel [28]. After 24 hours, humidity was reduced to 60% for the duration of each assay.

### Mortality

Dead flies were removed daily from all cages to avoid secondary inoculation of live flies by spores on the cuticles of the deceased flies and to record the numbers of dead females and males. Food plates were replaced with fresh ones daily.

#### Experiment 1: Effects of mating and sex on post-infection survival of *D. melanogaster* when inoculated with *B*. *bassiana* strain ARSEF 12460

Shahrestani et al. 2018 reported a sexual dimorphism in survival post infection with *B. bassiana* strain ARSEF 12460. We tested the hypothesis that mating affects the direction and magnitude of this sexual dimorphism and the hypothesis that female reproductive output is affected by mating frequency and infection. To test the effects of mating on post-infection survival, three mating conditions (virgin, mated, and cohabit) were established as follows. To ensure virginity of flies, on day five from egg, third instar larvae were transferred from rearing vials into individual straws using a paint brush. The straws had Cornmeal food on one end and were sealed with pipette tips on both ends. On day 12 from egg (2–3 days post eclosion), emerged flies were sexed while still in the straws, and the food in the straw was replaced. On day 16 from egg, flies were moved from straws to vials at densities of 30 flies/vial, using brief CO_2_ anesthesia. At this stage, the 30 flies in the vial were either all male or all female for the virgin groups, or half male and half female for the mated and cohabiting groups.

After 24 hours, on day 17 from egg, the flies were sprayed with fungus or control suspension. For each spray, 60 flies were anesthetized using carbon dioxide for 5 minutes on a CO_2_ pad to allow sorting by sex, then placed on ice for the ~2 minutes of the spray time. For the virgin groups, males and females were sprayed separately and kept in separate cages after the spray. For the mated groups, after 24 hours of mating in vials, males and females were sprayed separately and maintained in separate cages. Lastly, for the cohabiting groups, after 24 hours of mating in vials, males and females were sprayed together and cohabited in the same cages after the spray. Flies were in cages at densities of 60 same sex flies per cage, or 30 males and 30 females per cage. At least 257 flies per sex per treatment were tested. Food plates in cages were replaced daily, and eggs laid on the plates by cohabiting and 24-hour mated females were kept and incubated for 7 days until pupation, at which point the number of pupae were counted as a proxy of offspring count. Mortality and fecundity were followed for 21 days post spray. The entire experiment was replicated four times.

*Statistical analysis of Experiment 1*. All analyses were performed in the statistical software R [29–32]. To determine the effects of mating on the survival of female and male *D*. *melanogaster* when inoculated with the entomopathogenic fungus, *B*. *bassiana*, we used the Proportional Hazard model [33] with ‘Treatment’, ‘Sex’ and ‘Mating Status’ as main effects, and ‘Replicate’ and ‘Cage Number’ as random effect as well as a novel approach via Bootstrap for creating confidence intervals for survival probabilities. Before building the model, we tested the difference among all the replicates by Kaplan-Meier survival function [34] as well as log-rank test [35] and found no differences among the four replicates. To check the proportional hazards assumption, a scaled Schoenfeld residual was plotted and a test using the Schoenfeld residuals against the transformed time was conducted for each covariate. Because some covariates broke the assumption, a piecewise PH model was proposed, in which the time splitting points were selected by a stratified model. With additional interaction terms, a piecewise PH model was chosen using both likelihood test score and Akaike information criterion [36]. A likelihood ratio test was performed as suggested in [37], using log partial-likelihood with the frailty integrated out with the log-likelihood of a no-frailty model. Any non-significant random effect term was dropped. To detect any influential outliers, every observation was assessed by its delta-beta value for each predictor of the best model. Influential outliers were kept in the model as we had no reason to think they resulted from error. The PH model not only allows for hazard ratio estimates but also for predicted survival probabilities. We resampled observations of each fly group 1,000 times with replacement to construct 95% bootstrap intervals for a linear combination of the covariates in the model. To quantify the effect of mating on survival percent in the study, a piecewise Proportional Hazard model was proposed (Model 1).


coxph(time,event)=Fungal+Mated+Cohabit+Sex+Fungal×Sex+Mated×Sex+Fungal×Mated. (Model 1)

In this piecewise PH model, the 21-day experiment time was split into three time intervals: 0–5, 5–11, and 11–21. This transformed the covariates Fungal, Mated, and Cohabit to time-dependent ones. Each time-dependent covariate has different coefficient estimates over the time intervals:

$$h_{0-5}(x)=h_{o}exp[1.04I_{Fungal}+0.24I_{Mated}+1.03I_{Cohabit}+0.75I_{Male}-0.46I_{Fungal\;Male}-0.59I_{Mated\;Male}+0.76I_{Fungal\;Mated}]$$

$$h_{5-11}(x)=h_{o}exp[3.94I_{Fungal}+0.22I_{Mated}+1.52I_{Cohabit}+0.75I_{Male}-0.46I_{Fungal\;Male}-0.59I_{Mated\;Male}+0.76I_{Fungal\;Mated}]$$

$$h_{11-21}(x)=h_{o}exp[3.66I_{Fungal}-0.07I_{Mated}+0.83I_{Cohabit}+0.75I_{Male}-0.46I_{Fungal\;Male}-0.59I_{Mated\;Male}+0.76I_{Fungal\;Mated}]$$

The piecewise PH model gave us a measure of the effect of mating on survival at any time point and allowed for further visual inspection of the survival probabilities over the 21 days of the experiment. We also investigated the effects of infection and mating status on average offspring count per surviving female via the analysis of variance (ANOVA) [38]. We first calculated the number of offspring counts per surviving female fly and assessed the mean values of different groups of treatments and mating statuses. The analysis not only examined the main effects but also explored the interaction effects between treatments and mating statuses. Based on the final model, the means with standard errors and 95% confidence intervals were calculated. Also, post-hoc comparisons were performed to indicate which groups were significantly different from others.

#### Experiment 2: Sexual dimorphism in post-infection survival of *D*. *melanogaster* when inoculated with *B*. *bassiana* strain GHA

*B. bassiana* strain GHA was more readily available to us than strain ARSEF 12460 as it is a common biopesticide; therefore, GHA was the more appropriate choice of pathogen for testing the impacts of diet on sexual dimorphism in immunity. First we tested for sexual dimorphism post-infection with GHA, replicating and building upon our past work [7].

On day 12 from egg, flies were transferred out of rearing vials and into fresh vials in groups of 15 males and 15 females. To separate and count the flies by sex, we anesthetized them using a CO_2_ gun for 10 seconds and put them on the CO_2_ pad for 3 minutes. On day 17 from egg, flies were inoculated with GHA or control suspensions. For each spray, 60 flies were anesthetized with CO_2_ for 15 seconds to immobilize them and placed on Petri dishes on ice for the ~2 minutes of the spray time. Males and females were sprayed together and cohabited together after the spray. Mortality was followed for 21 days post spray. At least 500 flies were tested per sex per treatment. The experiment was replicated three times.

*Statistical analysis of Experiment 2*. Sexual dimorphism in survival after GHA inoculation was analyzed similarly as in Experiment 1 (see Model 2).


coxph(time,event)=Fungal+Sex (Model 2)

The time-dependent covariates have different coefficient estimates over the time intervals 0–8 days, 8–12 days and 12–21 days:

$$h_{0-8}(x)=h_{o}exp[4.18I_{GHA}-0.79I_{Male}]$$

$$h_{8-12}(x)=h_{o}exp[4.18I_{GHA}+0.36I_{Male}]$$

$$h_{12-21}(x)=h_{o}exp[4.18I_{GHA}+0.26I_{Male}]$$

#### Experiment 3: Effects of Cornmeal and Glucose diets on sexual dimorphism in post-infection survival of *D*. *melanogaster* inoculated with *B*. *bassiana* strain GHA

The Glucose diet is the ancestral diet of this population, with the Cornmeal diet being a relatively novel condition (note that Population C3 is a highly genetically variable outbred population with potential to respond to selective conditions). While experiment 2 above, which used Cornmeal diets, qualitatively replicated the results of Shahrestani et al. 2018 which used Glucose diets, the results different quantitatively. We directly tested the hypothesis that post-infection survival would be higher on the ancestral Glucose diet compared to survival on the Cornmeal diet. We also tested whether or not the timing of introduction of the Glucose diet (pre- or post- infection) affected post-infection survival.

On Day 12 from the egg, flies were transferred, in groups of 15 males and 15 females, to fresh vials containing an assigned diet: cornmeal (C) or glucose (G) (ingredients provided above). To separate and count the flies by sex, we anesthetized them using a CO_2_ gun for 10 seconds and put them on a CO_2_ pad for 3 minutes. On day 15 from egg, the flies were sprayed with either GHA and control suspensions. Sprayed flies were moved to cages and fed with either the C or G diets. The before and after spray diet combinations are summarized in S1 Table. Approximately 150 flies were sprayed per condition and kept in plexiglass cages (volume = 450 cm^3^). Dead flies were removed and sexed daily for 12 days (until age 27 days from egg). The experiment was replicated three times.

*Statistical analysis of Experiment 3*. Sexual dimorphism in survival after GHA inoculation and different diet conditions was analyzed similarly as in Experiment 1. A piecewise PH model was proposed (see Model 3).


$$coxph(time,\;event)=Fungal+Male+Diet+Fungal\times Male+Fungal\times Diet+Fungal\times Male\times Diet_{G/G}+Fungal\times Male\times Diet_{C/G}$$ (Model 3)

In this piecewise PH model, the 12-days after the spray (the days for which there is mortality data) were split into three time intervals: 0–3, 3–9, and 9–12. Each time-dependent covariate has coefficient estimates over these time intervals. The baseline *h*_*o*_ is females without infection. The estimated hazard ratio (*h*_*t*_(*x*)/*h*_*o*_) at a specific time point *t* between any other group *x* and the baseline can be obtained by the coefficient estimates:

$$h_{0-3}(x)=h_{o}exp[1.104I_{fungal}-0.193I_{female}+1.089I_{DietG/G}-0.796I_{DietG/C}+0.631I_{DietC/G}+0.533I_{fungal}\times I_{female}-1.476I_{fungal}\times I_{DietG/G}-0.232I_{fungal}\times I_{DietG/C}-15.140I_{fungal}\times I_{DietC/G}-1.732I_{fungal}\times I_{female}\times I_{DietG/G}+12.470I_{fungal}\times I_{female}\times I_{DietC/G}]$$

$$h_{3-9}(x)=h_{o}exp[3.879I_{fungal}-0.880I_{female}+1.098I_{DietG/G}+1.440I_{DietG/C}-0.510I_{DietC/G}+2.117I_{fungal}\times I_{female}-3.389I_{fungal}\times I_{DietG/G}-1.380I_{fungal}\times I_{DietG/C}-1.085I_{fungal}\times I_{DietC/G}+0.924I_{fungal}\times I_{female}\times I_{DietG/G}+0.694I_{fungal}\times I_{female}\times I_{DietC/G}]$$

$$h_{9-12}(x)=h_{o}exp[3.790I_{fungal}-0.005I_{female}-0.514I_{DietG/G}+0.392I_{DietG/C}-0.331I_{DietC/G}+0.098I_{fungal}\times I_{female}-0.926I_{fungal}\times I_{DietG/G}-0.204I_{fungal}\times I_{DietG/C}-2.919I_{fungal}\times I_{DietC/G}+0.865I_{fungal}\times I_{female}\times I_{DietG/G}+2.820I_{fungal}\times I_{female}\times I_{DietC/G}]$$

#### Experiment 4: Effects of yeast supplementation on sexual dimorphism in post-infection survival of *D*. *melanogaster* inoculated with *B*. *bassiana* strain GHA

The cornmeal and glucose diets used in Experiment 3 differ in their nutrients. The cornmeal diet has a much higher sugar content than the glucose diet. Comparatively, the overall protein to carbohydrate ratio for the glucose diet is 1:4, whereas cornmeal is 1:9, as calculated by the Drosophila Dietary Composition Calculator [18]. Yeast supplementation can provide a source of high protein. We tested the hypotheses that yeast supplementation of the cornmeal diet would help recover some of the post-infection survival differences seen on cornmeal versus glucose diets in Experiment 3, and that post-infection yeast supplementation would be more beneficial than pre- infection yeast supplementation because it would be less likely to trigger allocation of resources away from immune defense and to reproduction.

On Day 12 from egg, 15 males and 15 females were transferred to a fresh vial containing an assigned diet: cornmeal (C), glucose (G), or cornmeal with yeast supplement (CY). The cornmeal plus yeast supplement diet was made by adding a layer of yeast paste to the surface of the cornmeal diet. The yeast paste was made by using a ration of 1g of yeast to 5 mL of DI H_2_O. Then using a pipette, 5mg of yeast was added onto the surface of the cornmeal food in each vial, and 80mg of yeast was added onto the cornmeal food in each Petri dish, which gives the same amount of yeast per surface area for vials and plates.

Flies were handled in the same way as in Experiment 3. The experiment was replicated four times. The dietary conditions of Experiment 4 are shown in S2 Table.

*Statistical analysis of Experiment 4*. Sexual dimorphism in survival after GHA inoculation and different diet conditions was analyzed similarly as in Experiment 1. A piecewise PH model was proposed (see Model 4).


coxph(time,event)=Fungal+Male+Fungal×Male+Male×Diet+Fungal×Diet+Fungal×Diet×Male (Model 4)

The model was split into three time intervals: 0–4 days, 4–9 days, and 9–12 days after spray. Over these time intervals, the coefficient estimates were different:

$$h_{0-4}(x)=h_{o}exp[0.296I_{fungal}+0.104I_{Male}-0.385I_{fungal}\times I_{male}-0.367I_{male}\times I_{DietC/C}+0.783I_{male}\times I_{DietCY/CY}-0.195I_{male}\times I_{DietCY/C}{-0.183I}_{male}\times I_{DietG/G}+{46.450I}_{fungal}\times I_{DietC/C}-0.307I_{fungal}\times I_{DietCY/CY}-0.161I_{fungal}\times I_{DietCY/C}+{0.239I}_{fungal}\times I_{DietG/G}+0.453I_{fungal}\times I_{male}\times I_{DietC/C}-0.364{I_{fungal}\times I}_{male}\times I_{DietCY/CY}+1.156{I_{fungal}\times I}_{male}\times I_{DietCY/C}+0.287{I_{fungal}\times I}_{male}\times I_{DietG/G}]$$

$$h_{4-9}(x)=h_{o}exp[2.957I_{fungal}-0.831I_{Male}-0.030I_{fungal}\times I_{male}+0.959I_{male}\times I_{DietC/C}+{1.808I}_{male}\times I_{DietCY/CY}+1.245I_{male}\times I_{DietCY/C}+1.393I_{male}\times I_{DietG/G}+{1.266I}_{fungal}\times I_{DietC/C}{-0.600I}_{fungal}\times I_{DietCY/CY}+0.506I_{fungal}\times I_{DietCY/C}+{0.210I}_{fungal}\times I_{DietG/G}-0.704I_{fungal}\times I_{male}\times I_{DietC/C}-1.219{I_{fungal}\times I}_{male}\times I_{DietCY/CY}-0.822{I_{fungal}\times I}_{male}\times I_{DietCY/C}-2.907{I_{fungal}\times I}_{male}\times I_{DietG/G}]$$

$$h_{9-12}(x)=h_{o}exp[3.394I_{fungal}-1.272I_{Male}+1.488I_{fungal}\times I_{male}+1.659I_{male}\times I_{DietC/C}+{1.603I}_{male}\times I_{DietCY/CY}+1.793I_{male}\times I_{DietCY/C}{-12.280I}_{male}\times I_{DietG/G}+{1.260I}_{fungal}\times I_{DietC/C}-0.082I_{fungal}\times I_{DietCY/CY}+{0.524I}_{fungal}\times I_{DietCY/C}-0.247I_{fungal}\times I_{DietG/G}-1.823I_{fungal}\times I_{male}\times I_{DietC/C}-1.935{I_{fungal}\times I}_{male}\times I_{DietCY/CY}-1.920{I_{fungal}\times I}_{male}\times I_{DietCY/C}+10.430{I_{fungal}\times I}_{male}\times I_{DietG/G}]$$

#### Experiment 5: Effects of varying levels of yeast supplementation on sexual dimorphism in post-infection survival of *D*. *melanogaster* inoculated with *B*. *bassiana* strain GHA

In Experiment 4 we showed that infected flies kept on yeast-supplemented cornmeal diets had better post-infection survival than flies kept on cornmeal diets without yeast. Next we tested the hypothesis that the amount of yeast affects this outcome, with more yeast supplementation further increasing the benefit to post-infection survival.

Flies were reared on cornmeal diet, then on day 12 from egg, they were moved to fresh vials containing cornmeal diet at densities of 15 male and 15 females per vial. On day 15, flies were sprayed as in Experiment 3. After the spray, they were moved to cages and fed with Petri Dishes of food that differed in their level of yeast supplementation. One condition used the same supplementation as in Experiment 4 (C/CY1.0), a second condition used 0.5x the amount of yeast (C/CY0.5), and a third condition used 1.5x the amount of yeast (C/CY1.5). A control condition used cornmeal diet without supplementation (C/C). The yeast levels were adjusted by altering the amount of yeast suspended in DI water, such that the same total volume of suspension was added to the food dishes.

*Statistical analysis of Experiment 5*: Sexual dimorphism in survival after GHA inoculation and different diet conditions was analyzed similarly as in Experiment 1. A piecewise PH model was proposed (see Model 5).


coxph(time,event)=Fungal+Male+Fungal×Male+Fungal×Cornmeal+Fungal×Male×Cornmeal+Fungal×Male×CY0.5 (Model 5)

The model was split into three time intervals: 0–5 days, 5–8 days, and 8–12 days after spray. Each time interval has a model formula given below. Over these time intervals, the coefficient estimates were different:

$$h_{0-5}(x)=h_{o}exp[0.886I_{fungal}+0.063I_{female}+0.485I_{DietC}+0.178I_{DietCY0.5}+0.107I_{DietCY1.0}-0.224I_{fungal}\times I_{female}-0.571I_{fungal}\times I_{DietC}+0.939I_{fungal}\times I_{female}\times I_{DietC}+0.053I_{fungal}\times I_{female}\times I_{DietCY0.5}]$$

$$h_{5-8}(x)=h_{o}exp[1.343I_{fungal}+0.134I_{female}-1.389I_{DietC}-0.256I_{DietCY0.5}-0.255I_{DietCY1.0}+0.988I_{fungal}\times I_{female}+2.396I_{fungal}\times I_{DietC}+0.207I_{fungal}\times I_{female}\times I_{DietC}+0.769I_{fungal}\times I_{female}\times I_{DietCY0.5}]$$

$$h_{8-12}(x)=h_{o}exp[2.776I_{fungal}+0.028I_{female}-0.486I_{DietC}+0.154I_{DietCY0.5}-0.238I_{DietCY1.0}+0.090I_{fungal}\times I_{female}+1.318I_{fungal}\times I_{female}-0.342I_{fungal}\times I_{female}\times I_{DietC}+0.047I_{fungal}\times I_{female}\times I_{DietCY0.5}]$$

## Results

### Mating status affects survival after inoculation with *B*. *bassiana* ARSEF 12460 in both male and female *D*. *melanogaster* (Experiment 1)

**Fig 1:**
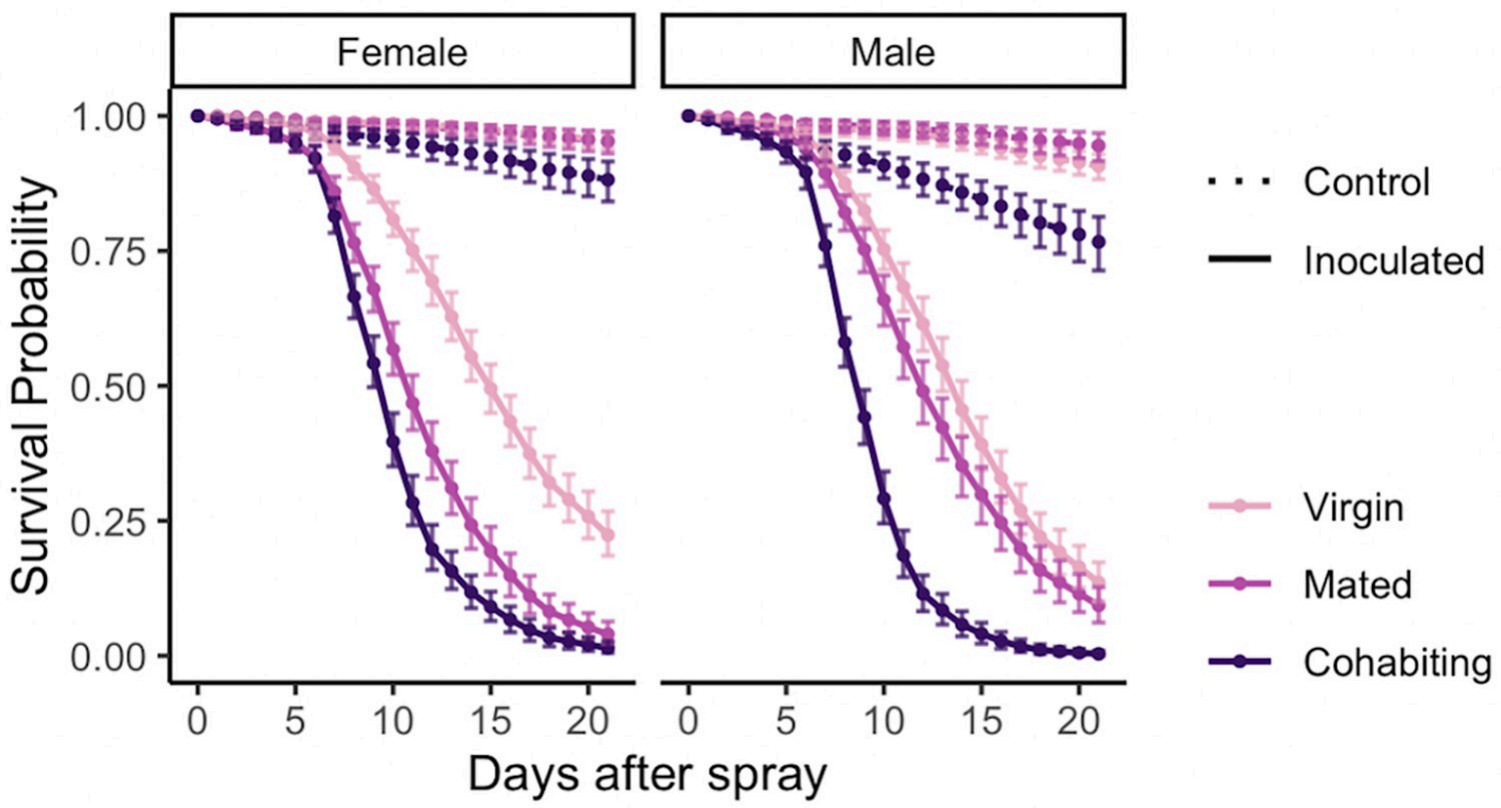
Survival post inoculation with *Beauveria bassiana* is affected by mating status in both males and females (Experiment 1). Flies were sprayed with fungal suspension (*n* = 1658, solid lines) or with a control, fungus-free suspension (*n* = 1626, dashed lines) at age 17 days from egg, and survival was followed for 21 days. In both females and males, virgin flies (pink) has the highest post-inoculation survival, followed by lower survival in flies that mated for a single day prior to inoculation (light purple), and lowest survival in flies that cohabited with the other sex before and after inoculation (dark purple) (see S1 Table for statistical analysis). This figure shows model estimates for survival proportions, which are obtained from the raw survival data (S3 Table, S1 Fig).

Inoculated flies died faster than control flies, suggesting the effectiveness of *B*. *bassiana* infection (Fig 1, raw data in S3 Table). Among infected females, virgins survived better than mated and cohabiting females in all time intervals (Fig 1, S1 Fig, S4 Table), and mated females survived better than cohabiting females (Fig 1, S1 Fig, S4 Table). For males, the same pattern was true in the 5–11 and 11–21 day intervals (Fig 1, S1 Fig, S4 Table). Even among control (uninfected) females and males, virgins showed a significantly higher survival than cohabiting flies in the 5–11 and 11–21 intervals (Fig 1, S1 Fig, S4 Table).

### Survival after inoculation with *B*. *bassiana* ARSEF 12460 is sexually dimorphic, but the direction of the dimorphism depends on mating status (Experiment 1)

Sexual dimorphism in post infection survival was observed in all three mating statuses. Among infected cohabiting flies, females showed a better survival than males (*p*-value = 0.0130, S1 Fig, S5 Table). The same trend was observed in virgin flies (*p*-value = 0.0016, S1 Fig, S5 Table). However, the trend was reversed in mated flies, where the males survived better than females (*p*-value = 0.0084; S1 Fig, S5 Table).

### Reproductive output is affected by infection and mating status (Experiment 1)

**Fig 2:**
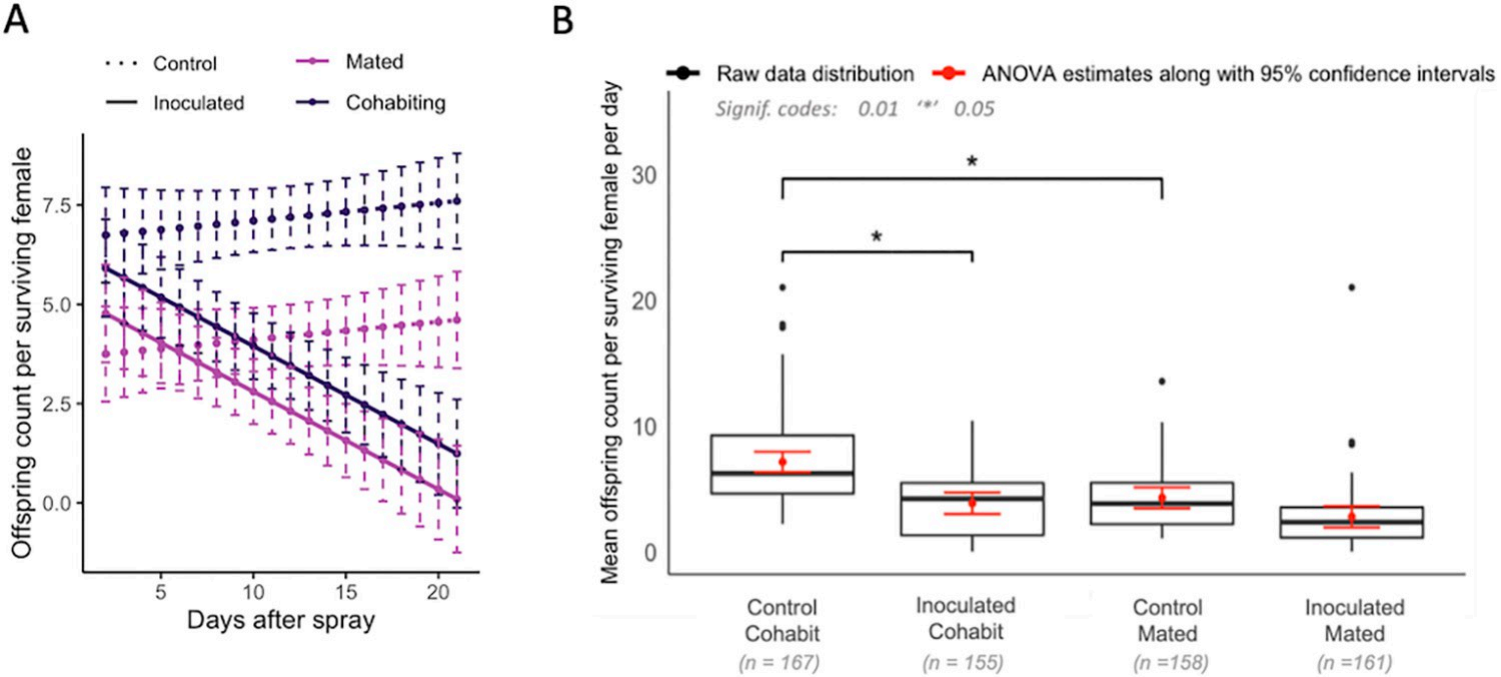
Reproductive output of mated and cohabiting females from both control and fungal inoculated conditions (Experiment 1). (A) Analysis of variance for the offspring counts produced by control (dashed lines) and fungal inoculated (solid lines) females at two different mating statuses, cohabiting (dark purple) and one-day mated (light purple). The data points are plotted for 95% confidence intervals. The fungal inoculated groups showed a lower offspring count in comparison to the control groups (p-value = <0.0001). The mated groups showed a lower offspring count in comparison to the cohabiting group in control treatment (p-value = <0.0001). There was an interaction effect between treatment (fungal/control) and mating status (cohabit/mated) with a p-value of 0.0373. (B) Box plot shows the raw data distribution of offspring counts per surviving female and predicted mean value for cohabiting and mated flies. Black dots represent the outliers while red lines represent the 95% confidence intervals. Control cohabiting females have a higher offspring count than the fungal cohabiting females (p-value = 0.0120) whereas there was no significant difference between offspring counts in control mated and fungal mated groups (p-value = 0.0870). Control cohabiting females had a significantly higher offspring count than the control mated (p-value = 0.0169). However, inoculated cohabiting and inoculated mated groups did not show a significant difference in offspring counts (p-value: 0.1771). The control cohabiting females (predicted mean: 7.124) have a lower offspring counts than fungal cohabiting females (predicted mean: 3.844) and the same was seen in control mated (predicted mean: 4.276) and fungal mated females (predicted mean: 2.772).

Mated and cohabiting females that were inoculated had offspring counts that declined with age over the span of 21 days post inoculation (Fig 2A). As the flies aged, the fungal inoculated groups showed lower offspring counts than the controls, as indicated by the *p*-value of <0.001 in the interaction between days and treatment (S6 Table). In the control groups, females that cohabited with males had more offspring than females that mated only for one day, but in the fungal inoculated groups, the cohabiting and single-day mated females had the same numbers of offspring measured by counting pupal casing (Fig 2B).

### Survival after inoculation with *B*. *bassiana* GHA is sexually dimorphic, but only for the first few days after inoculation (Experiment 2)

**Fig 3:**
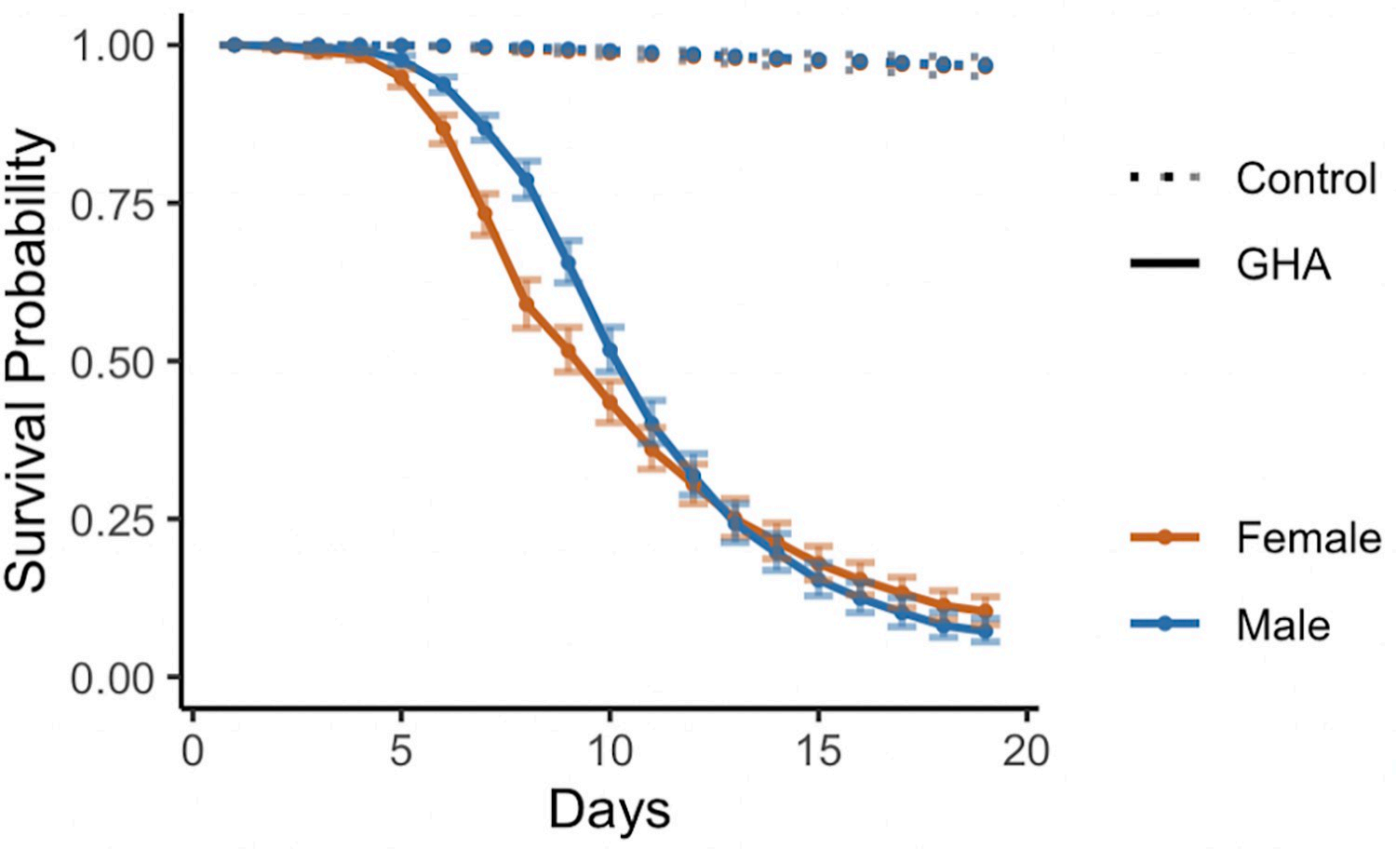
Sexual dimorphism in survival of *D*. *melanogaster* after inoculation with *B*. *bassiana* strain GHA (Experiment 2). Inoculated males (blue) survived better than inoculated females (orange) until day 12 post spray. See S5 Table for statistical analysis. This figure shows model estimates with 95% Bootstrap confidence intervals from the raw data shown in S7 Table.

Shahrestani et al. [7] reported that when *D*. *melanogaster* Population C3 (same outbred population used here) was inoculated with GHA under cohabiting conditions, males survived better than females in the ten days post-inoculation. Their study did not extend beyond 10 days. Replicating their study, we found that after inoculation with GHA, males survived better than females for the first 12 days after inoculation, but thereafter the female and male survivals converged for the remainder of our 21-day study (Fig 3, raw data in S7 Table, analysis in S8 Table).

### Introduction of Glucose diet pre- and post- inoculation with *B*. *bassiana* GHA affects survival in sex-specific ways (Experiment 3)

**Fig 4:**
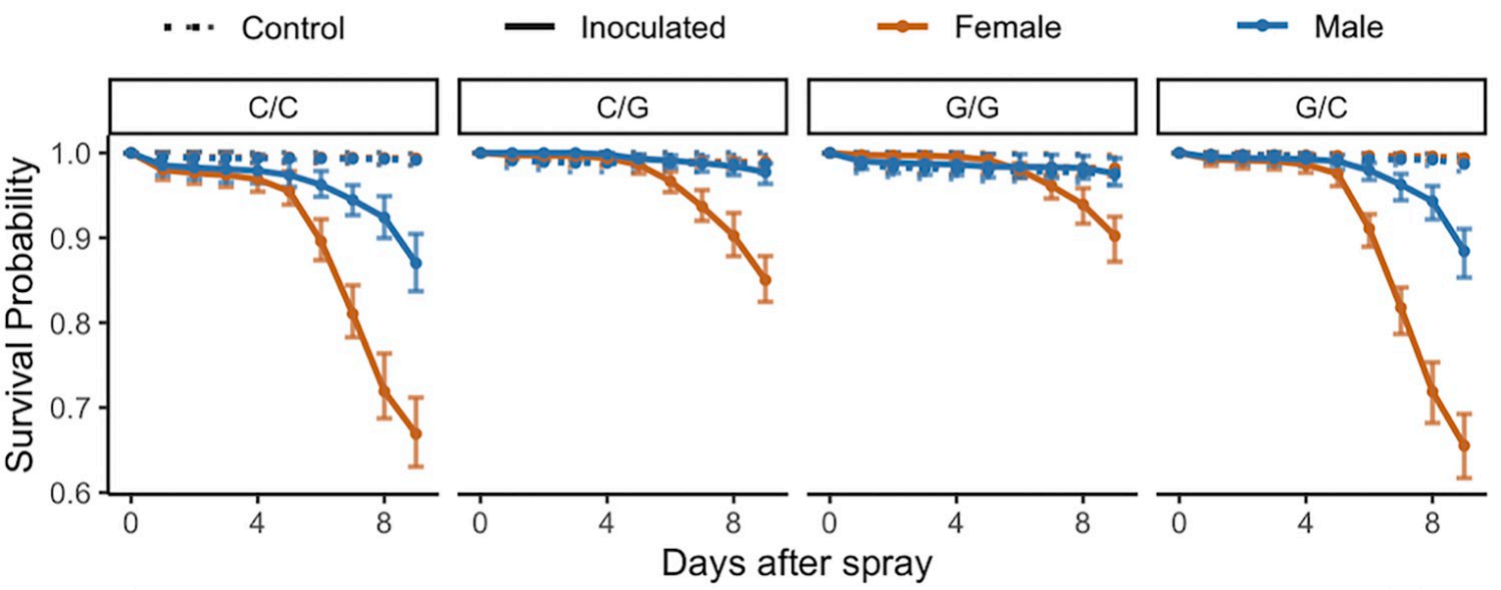
Effects of diet on post-inoculation survival of *D*. *melanogaster* inoculated with *B*. *bassiana* strain GHA (Experiment 3). Inoculated flies (Solid lines) had an overall lower survival than control flies (Dashed lines) under all dietary conditions (pre/post): cornmeal/cornmeal (C/C), cornmeal/glucose (C/G), glucose/glucose (G/G), and glucose/cornmeal (G/C). There was no sexual dimorphism observed among control flies, but among inoculated flies males (blue) survived better than females (orange). The timing of introduction of a glucose diet affected post inoculation survival. For statistical analysis of these data see S4 and S5 Tables. The figure shows model estimates with 95% Bootstrap confidence intervals from the raw data shown in S9 Table.

Control flies survived better than inoculated flies regardless of diet (Fig 4). Flies that received glucose diets after inoculation with *B*. *bassiana* survived better than those that received cornmeal diets (Fig 4, raw data in S9 Table). The time of introduction of the glucose diet impacted the survival of flies, especially in males (Fig 4). S10 Table summarizes the statistical comparisons of hazard ratios among males and females of control and inoculated groups under the various dietary conditions. Among uninfected controls, there was no sexual dimorphism in survival observed under any dietary condition. Among inoculated flies, when the post-inoculation diet was cornmeal, regardless of whether the pre-inoculation diet was cornmeal or glucose, females had higher hazard than males (survived worse) in the 3–9 day interval post inoculation, but male and female hazards were the same in the 0–3 and 9–12 day intervals post inoculation (S10 Table). When flies were given a glucose diet post inoculation, regardless of whether the pre-inoculation diet was cornmeal or glucose, males survived better than females in both the 3–9 and 9–12 day intervals post inoculation (S10 Table). S11 Table summarizes the effects of diet on survival in control and inoculated flies relative to survival on Cornmeal diets. In both females and males, receiving a glucose diet improved post-inoculation survival in most of the time intervals (S11 Table).

### Yeast supplementation pre- and post- inoculation with *B*. *bassiana* GHA positively impacts survival, but not as much as the Glucose diet (Experiment 4)

**Fig 5:**
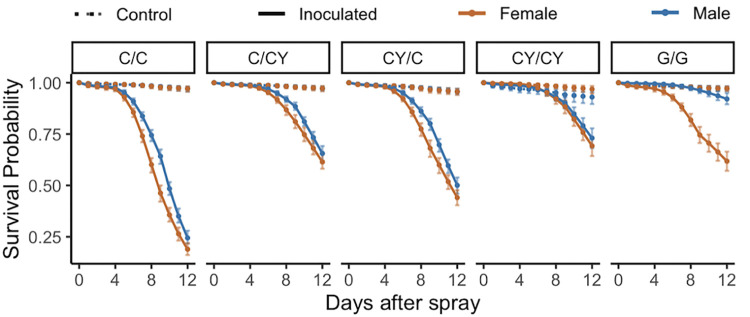
Yeast supplementation affects sexual dimorphism in survival of *D*. *melanogaster* inoculated with *B*. *bassiana* strain GHA (Experiment 4). Three days prior to control spray (dashed lines) or fungal spray (solid lines), flies were given cornmeal (C), cornmeal with yeast supplement (CY), or glucose (G) diets (marked before the dash in the figure headings). After the spray, which was on day 15 from egg, flies were kept on one of the three types of diets (C, CY, or G, marked after the dash in the figure headings). Yeast supplementation improved survival of inoculated males (blue) and females (orange). When yeast supplementation was provided after the inoculation, sexual dimorphism was ablated. But there was sexual dimorphism among control flies when yeast supplementation was provided both before and after the spray. See S6 Table for analysis of these results. The figure shows model estimates for the raw data shown in S12 Table.

In all dietary conditions, control males and females showed a higher survival than the fungal inoculated males and females (Fig 5, raw data in S12 Table). Cornmeal fed flies who received cornmeal diet before and after the spray had the lowest post-inoculation survival (Fig 5). Flies that were fed with cornmeal before spray, but yeast supplemented cornmeal after the spray showed a rescue in survival in both males and females (Fig 5, S13 Table). Flies fed with yeast supplemented cornmeal before inoculation and cornmeal after the inoculation showed lower survival than flies that received yeast supplementation before and after inoculation (Fig 5, S13 Table). The highest survival was seen in flies that were given Glucose diets before and after inoculation, followed by the condition in which flies received yeast supplemented cornmeal diet before and after inoculation (Fig 5). Yeast supplementation affected sexual dimorphism in surviving inoculation (Fig 5, S14 Table). When yeast supplementation was provided after inoculation, it ablated the sexual dimorphism in survival of inoculation (S14 Table). However, when yeast supplementation was provided before inoculation, but not after, sexual dimorphism in survival was present in the 3–9 day time intervals, similarly to the control cornmeal diet (S14 Table). On the glucose diet, males survived better than females in both the 3–9 day and 9–12 day time intervals (S14 Table). Under uninfected conditions, when yeast supplementation was provided before and after the spray, male survival was negatively impacted relative to female survival (S14 Table).

### The amount of supplemental yeast added to the diet impacts survival post inoculation with *B*. *bassiana* GHA (Experiment 5)

**Fig 6:**
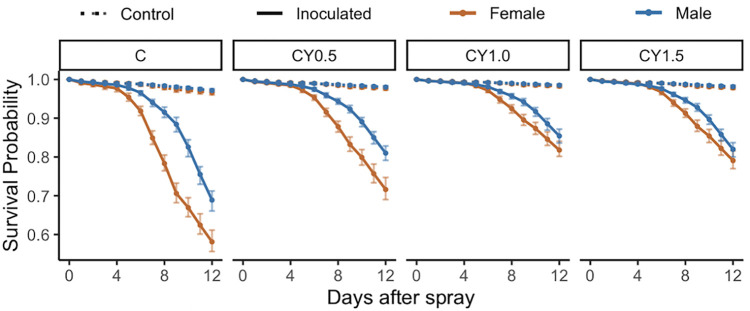
Level of yeast supplementation affects sexual dimorphism in survival when *D*. *melanogaster* are inoculated with *B*. *bassiana* GHA (Experiment 5). All flies were reared on cornmeal diets. After the sprays, flies received cornmeal diets supplemented with varying levels of yeast. See S7 and S8 Tables for statistical analysis of these results. The figure shows model estimates and 95% confidence intervals for the raw data shown in S15 Table.

Control flies survived better than inoculated flies regardless of diet and did not present sexual dimorphism (Fig 6, raw data in S15 Table). The addition of yeast supplement after inoculation, when done at the same dose as in Experiment 4 (1 g yeast/ 5mL DI water) improved survival of both males and females relative to flies maintained on cornmeal-only diet (Fig 6, S16 Table) similar to the results of Experiment 4. At half the amount of yeast supplement, post inoculation survival was still improved relative to the cornmeal-only diet, but survival was still significantly lower than with the higher dose of yeast supplementation (S16 Table). When 1.5x the amount of yeast supplement was added relative to the amount in Experiment 4, the impact on post inoculation survival did not change (S16 Table). Sexual dimorphism in survival post-inoculation was present in the 0–5 and 5–8 day intervals for the cornmeal-only and lowest level of yeast supplementation (S17 Table), but the sexual dimorphism was ablated in the 0–5 day interval at higher levels of yeast supplementation (S17 Table).

## Discussion

In this study we have uncovered some of the complexity in how post-infection survival is affected by sex, mating, and diet. Replicating the results of Shahrestani et al. [7] we showed that the *D*. *melanogaster* Population C3 response to infection with the fungus *Beauveria bassiana* strain GHA is sexually dimorphic with males surviving infection better than females. We additionally found that this sexual dimorphism in immune defense begins to disappear around 12 days post inoculation, such that females and males have the same probability of survival from days 12–21 post inoculation. As such, the existence of post-infection sexual dimorphism in survival is affected by the time of observation. With a second pathogen, *Beauveria bassiana* strain ARSEF 12460 we found that Population C3 males were more susceptible to infection than females and this sexual dimorphism persisted through the 21 days of the experiment. This result is contrary to our prior work with this pathogen, in which females were more susceptible than males, but in a different fly genotype called Canton S [7]. The impact of host genotype on the direction of sexual dimorphism in post-infection survival was also seen in our prior work in which we compared ~300 recombinant inbred lines from the DSPR and saw that some fly lines had no sexual dimorphism in post-infection survival, and when there was sexual dimorphism, the magnitude and direction of it (which sex was more susceptible) depended on fly genotype [8].

Aside from using a different fly line than ours, the Shahrestani et al. [7] study used only mated flies (akin to our “cohabiting” condition), while we used virgin, singly mated, and multiply mated (cohabiting) flies. Our results show that mating status affects the direction of sexual dimorphism in immune defense. Among cohabiting flies, where males continued pre-copulation behaviors, like competition and courtship, males were more susceptible to infection than females. However, when flies were mated for only 24-hours prior to inoculation, females were more susceptible to infection than males. Yet when virgin flies were compared, males were again more susceptible than females. We found that the mating-immunity trade-off is at least in part affected by the frequency of mating, instead of being an inherent component of male and female biology.

Mating frequency not only affected the direction of sexual dimorphism in immune defense, it also affected the total post-infection survival; both males and females survived infection better when they were virgins, followed by more deaths if they had mated, and even more deaths if they cohabited. It is previously documented that mating suppresses immune defense in insects [17, 39–41]. In female *D*. *melanogaster*, bacterial and fungal infections lower fecundity [21, 23, 25, 42, 43]. The negative impacts of mating on immunity, and of infection on fecundity, suggest a possibility that limited resources are being shared among these traits. We tested for differences in resource allocation in females by counting the number of offspring produced with and without infection in the mated and cohabiting groups.

Infected females produced fewer offspring than control females, suggesting that there is a reproductive cost to infection. Under uninfected conditions, females that cohabited with males produced more offspring than females that mated only for one day, which was expected due to the continued mating in the cohabiting condition. In uninfected cohabiting females, we did not see an age-specific decline in fecundity in the 21 days of this assay, which is not surprising in this particularly robust outbred population. But the same stable daily fecundity was also seen in females that mated only for one day. One potential explanation for this is that female *D*. *melanogaster* can store sperm post-copulation for delayed release [44]. Under infected conditions, there was a decline in fecundity over the 21 day assay in both cohabiting and one-day mated females. But in these infected conditions, cohabiting and one-day mated females laid indistinguishable numbers of eggs, which may suggest that the increased infection susceptibility of cohabiting females was not due to increased egg laying. However, it is possible that, due to infection, the numbers of eggs laid were too low for differences to be picked up with our statistical analyses.

We did not measure male reproductive output. However, other factors, outside of resource allocation to reproduction could potentially affect a trade-off of reproduction with immune defense. For example, mating itself is energetically costly, and pre-copulatory behaviors, such as competition and courtship give rise to energy expenditure. In our study, cohabiting flies were maintained in population cages with equal sex ratios. The stress and investment for successful mating could have affected male susceptibility to infection. Note that the fungus *B*. *bassiana* is not itself transmitted by mating.

Taken together, Experiments 1 and 2 compared with our past studies [7, 8] suggest that post-infection survival in *D*. *melanogaster* is affected by fly genotype, fungal strain, and mating status. But going back to the opposite result we obtained for sexual dimorphism in response to infection with ARSEF 12460 in our study compared to Shahrestani et al. [7], we note the different diets used in these studies. Laboratories that use *D*. *melanogaster* vary greatly in the diets that they provide to their fly populations [18], and the impacts of diet on *D*. *melanogaster* can also be sexually dimorphic [e.g. 45]

We found that *D*. *melanogaster* Population C3 survived infection better when placed on a glucose diet as opposed to a cornmeal diet. One possible explanation for this observation is that Population C3 was better adapted to the glucose diet, which it received for more total number of generations before being moved to a cornmeal diet in its recent evolutionary history. However, it is additionally possible that the higher protein to carbohydrate ratio of the glucose diet is responsible for the improvements in post-infection survival, in which case yeast-supplementation of the cornmeal diet should produce a similar improvement. Indeed, when flies were given a yeast supplement after infection, they had higher survival of the infection. While the timing of introduction of the yeast mattered, even yeast supplementation prior to infection improved post-infection survival when compared to cornmeal diets without any yeast supplementation. And even low amounts of yeast supplementation improved survival of infection. Increasing the amount of yeast led to even greater benefits for survival but only up to intermediate yeast levels, beyond which additional yeast did not lead to additional benefits. Notably, sexual dimorphism in immune defense was larger on the glucose diet than on yeast-supplemented cornmeal diets despite total survival being higher in both of these conditions when compared to the sugar-rich cornmeal diet. This suggests that protein levels are not the only factor involved, and other dietary components affect sexual dimorphism in response to infection.

The relationships among sex, mating and immunity can have evolutionary consequences. For example, female post-mating immune suppression could lead to reallocation of limited resources to immediate reproduction, increasing the paternity of the most recent sexual partner, potentially affecting sexual conflict [9, 17, 40]. Pleiotropic relationships among immunity and mating have the potential to alter or limit the function and evolution of overall immune defense. Our results show that these relationships are influenced by diet; as such, the evolutionary landscape of sexual selection and sexual conflict affecting these traits is guided by dietary condition.

## Supporting information

S1 Fig Sexual dimorphism in survival of *D*. *melanogaster* inoculated with *B*. *bassiana* strain ARSEF 12460 is affected by mating status (Experiment 1). Female (orange) and male (blue) survival after control spray (dashed lines) and fungal spray (solid lines) is shown for cohabiting flies, virgin flies, and mated flies which mated for only 24 hours. Survival was followed for 21 days after the spray. Sample sizes per treatment are provided in the legend. The top graphs show model estimates for survival proportions, using four replicates of raw data with 95% Bootstrap confidence intervals. See S1 and S2 Tables for statistical analysis of this data. Bottom graphs show the raw data plotted for the four replicates of each treatment and the means. For cohabiting and virgin flies, females had better survival than males after inoculation. For mated flies, this trend was reversed. In both females and males, virgin survival was higher than mated survival, which was itself higher than survival under cohabiting conditions.
(TIF)

S1 Table Dietary treatments of Experiment 3. For each dietary condition, half of the flies were inoculated and half were treated as controls. All flies were reared on a cornmeal diet until age 12 from egg. Then some flies received cornmeal and some received a glucose diet. After flies were sprayed at age 15 days from egg, some flies received cornmeal and some glucose diet.
(PDF)

S2 Table Dietary treatments of Experiment 4. For each dietary condition, half of the flies were inoculated and half were treated as controls. All flies were reared on a cornmeal diet until age 12 from egg. Then the specific dietary conditions were applied.
(PDF)

S3 Table Raw Data from Experiments 1. All of the data from Experiment 1 are shown. The control treatment is a spray without any pathogen and the Inoculated treatment is sprayed with *Beauveria bassiana* ARSEF 12460. Flies had three mating statuses, virgin, mated (which mated for one day prior to the spray) and cohabit (which mated before and after the spray). Both females (F) and males (M) were tested in cages, with sexes separated or mixed. Separate cages within the same replicate are numbered. Four replicates were done for each condition. The assay continued for 21 days and the deaths per day were recorded (day 1 data were removed due to handling loss). The starting number of flies in each cage are shown in the survival column. Daily offspring count is shown for groups of mated and cohabiting females.
(XLSX)

S4 Table Hazard ratios and *p*-Values when comparing mating statuses in both control and fungal inoculated *Drosophila melanogaster* (Experiment 1). Hazard ratios and *p*-Values are presented in the time intervals of 0–5, 5–11, and 11–21 days post inoculation.
(PDF)

S5 Table Hazard ratios and *p*-Values when comparing males and females under different mating statuses in both control and fungal inoculated *Drosophila melanogaster* (Experiment 1). Hazard ratios and *p*-values are presented for days 0–21 post inoculation.
(PDF)

S6 Table Table representing the interaction between days and treatment between the Control and Fungal inoculated reproductive output of the flies (Experiment 1). Significantly smaller *p*-value of Treatment (<0.0001) indicates that the data strongly supports the difference on offspring counts between the fungal inoculated groups and the controls. The data also strongly supports that cohabiting and mated groups differ in their offspring counts (*p*-value = <0.0001). However, inoculated cohabiting and inoculated mated groups did not show a significant difference in offspring counts (see Fig 2 description, p-value: 0.1771). * p-value ≤ 0.05, *** p-value ≤ 0.001.
(PDF)

S7 Table Raw data for Experiment 2. All of the data from Experiment 2 are shown. The control treatment is a spray without any pathogen and the inoculated treatment is sprayed with *Beauveria bassiana* GHA. All flies were in cohabiting conditions. The assay continued for 19 days, and daily deaths are shown (day 1 data is removed due to handling loss). The starting number of flies in each cage are shown in the “initial density” column. The experiment was repeated three times, each time with two replicate cages per condition (see cage number column).
(XLSX)

S8 Table Hazard ratios and *p*-values for males vs females in GHA inoculated flies (Experiment 2). The hazard ratios indicate the risk of male flies dying post inoculation in comparison to the female flies. The data strongly supports that there is sexual dimorphism in post inoculation survival in the 0–8 and 8–12 age intervals. However the *p*-value in the 12–21 days interval suggests there is no sexual dimorphism in survival in this interval.
(PDF)

S9 Table Raw data for Experiment 3. All data from Experiment 3 are shown. The control treatment received a spray without pathogen and the fungal treatment received a spray with *Beauveria bassiana* GHA. All flies were in cohabiting conditions. Flies received four diet conditions as explained in S1 Table, receiving cornmeal (C) or glucose (G) before and/or after the spray. The assay continued for 9 days. Daily deaths are shown for both males and females. The starting number of flies in each cage are shown in the “initial density” column. The experiment was twice replicated.
(XLSX)

S10 Table Diet affects sexual dimorphism of *D*. *melanogaster* inoculated with *B*. *bassiana* strain GHA in a sex-specific manner (Experiment 3). There was no sexual dimorphism in survival among the control treatments, regardless of diet. However, among fungal inoculated flies, males survived better than females in some age intervals on every diet. The timing of introduction of a glucose diet has an impact on the Hazard ratio among inoculated flies. Statistically significant differences are in bold.
(PDF)

S11 Table Effects of dietary condition and infection status on male and female survival in different age groups (Experiment 3). Effects of inoculation and diet changes on males and females compared to control males on cornmeal diet.
(PDF)

S12 Table Raw data for Experiment 4. All data for Experiment 4 are shown. The control treatment received a spray without pathogen and the fungal treatment received a spray with *Beauveria bassiana* GHA. All flies were in cohabiting conditions. Flies received five diet conditions as explained in S2 Table, receiving different pre- and post- inoculation diets of cornmeal (C), cornmeal with yeast supplement (CY), or glucose (G). The assay continued for 12 days. Daily deaths are shown for both males and females. The starting number of flies in each cage are shown in the “initial density” column. The experiment was replicated three times and each time two technical replicates in separate cages were done for each condition (labeled alpha and beta in the cage number column).
(XLSX)

S13 Table Effect of diet on survival of males and females combined when inoculated with *B*. *bassiana* GHA (Experiment 4). Hazard ratios and *p*-values are presented from 0–4, 4–9 and 9–14 days post inoculation.
(PDF)

S14 Table Yeast supplementation affects sexual dimorphism in surviving infection (Experiment 4). Whenever yeast supplementation was provided after inoculation, it reduced the sexual dimorphism in survival. When yeast supplementation was provided before and after the spray, there was sexual dimorphism among uninfected flies. The hazard ratio, showing difference in hazard between males and females is largest for flies that received the glucose diet.
(PDF)

S15 Table Raw data for Experiment 5. All data from Experiment 5 are shown. The control treatment received a spray without pathogen and the fungal treatment received a spray with *Beauveria bassiana* GHA. All flies were in cohabiting conditions. Flies received four diet conditions: cornmeal (C) and cornmeal supplemented with three levels of yeast (CY0.5, CY1.0, CY1.5). The assay continued for 12 days. Daily deaths are shown for both males and females. The starting number of flies in each cage are shown in the “initial density” column. The experiment was replicated twice each time with two technical replicates per condition in separate cages (labeled alpha and beta in the cage number column).
(XLSX)

S16 Table Intermediate levels of yeast supplementation improved survival (Experiment 5). Pairwise comparisons of survival are shown, comparing the control (no yeast) condition to the lowest yeast level, comparing the lowest and intermediate yeast levels to each other, and comparing the intermediate and high yeast levels to each other. In both males and females, survival is improved by low and intermediate levels of yeast supplementation.
(PDF)

S17 Table There was no sexual dimorphism in survival among control flies (Experiment 5). When flies were inoculated with fungus, there was sexual dimorphism on all diets, but the age intervals and magnitudes of this dimorphism changed with the level of yeast supplementation. When there is no yeast supplement or a little amount of supplement, the dimorphism starts at earlier ages than with higher levels of yeast supplement.
(PDF)
