# Supplementary figures and images for: Immune defense in Drosophila melanogaster depends on diet, sex, and mating status
Source: PLoS One. 2023 Apr 13;18(4):e0268415. doi: 10.1371/journal.pone.0268415 (PMC10101424; doi:10.1371/journal.pone.0268415)

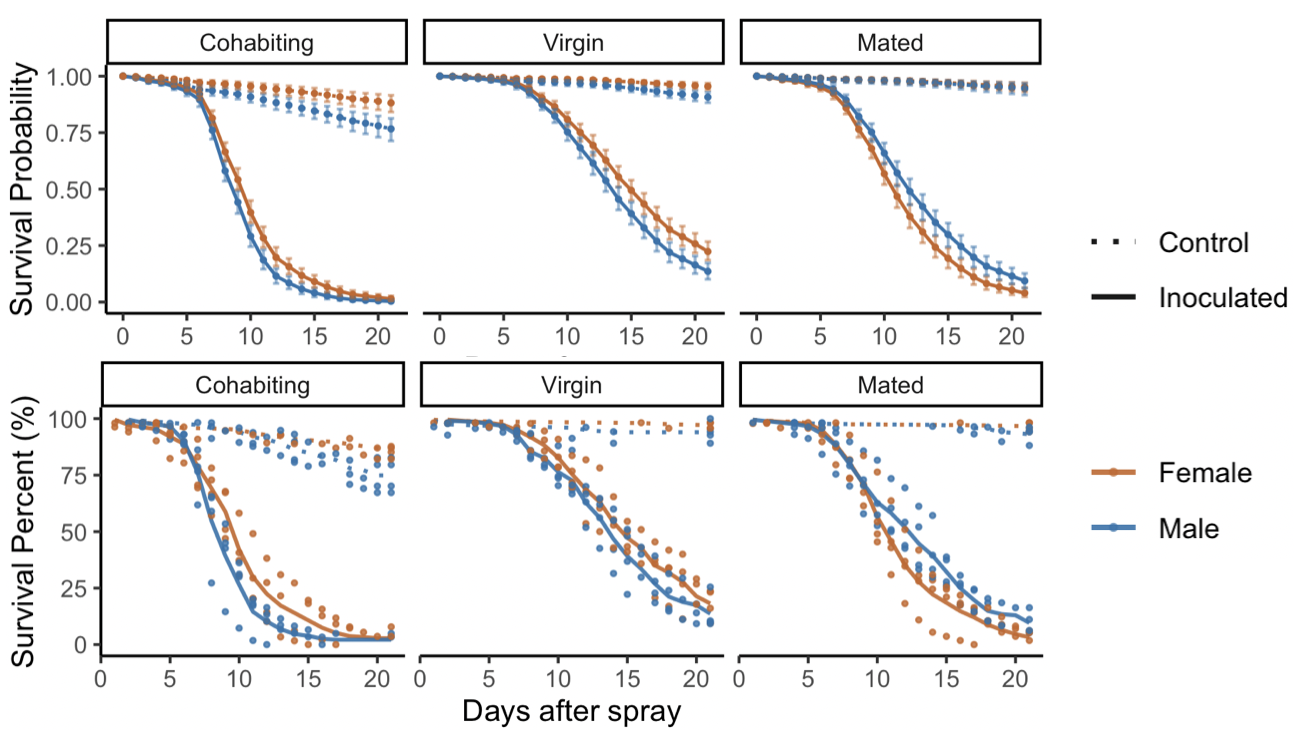

Supplement: S1 Fig — Female (orange) and male (blue) survival after control spray (dashed lines) and fungal spray (solid lines) is shown for cohabiting flies, virgin flies, and mated flies which mated for only 24 hours. Survival was followed for 21 days after the spray. Sample sizes per treatment are provided in the legend. The top graphs show model estimates for survival proportions, using four replicates of raw data with 95% Bootstrap confidence intervals. See S1 and S2 Tables for statistical analysis of this data. Bottom graphs show the raw data plotted for the four replicates of each treatment and the means. For cohabiting and virgin flies, females had better survival than males after inoculation. For mated flies, this trend was reversed. In both females and males, virgin survival was higher than mated survival, which was itself higher than survival under cohabiting conditions. (TIF) [file pone.0268415.s001.tif]
