# Supplementary material for: Immune defense in Drosophila melanogaster depends on diet, sex, and mating status
Source: PLoS One. 2023 Apr 13;18(4):e0268415. doi: 10.1371/journal.pone.0268415 (PMC10101424; doi:10.1371/journal.pone.0268415)
Supplement: S1 Table — For each dietary condition, half of the flies were inoculated and half were treated as controls. All flies were reared on a cornmeal diet until age 12 from egg. Then some flies received cornmeal and some received a glucose diet. After flies were sprayed at age 15 days from egg, some flies received cornmeal and some glucose diet. (PDF) [file pone.0268415.s002.pdf]

Table S1. Dietary treatments of Experiment 3.

For each dietary condition, half of the flies were inoculated and half were treated as controls. All flies were reared on a cornmeal diet until age 12 from egg. Then some flies received cornmeal and some received a glucose diet. After flies were sprayed at age 15 days from egg, some flies received cornmeal and some glucose diet.

Treatment name on Fig. 4	Age 12 - 14 (before spray)	Age 15 - 27 (after spray)
C/C	Cornmeal	Cornmeal
C/G	Cornmeal	Glucose
G/G	Glucose	Glucose
G/C	Glucose	Cornmeal
