# Supplementary material for: Immune defense in Drosophila melanogaster depends on diet, sex, and mating status
Source: PLoS One. 2023 Apr 13;18(4):e0268415. doi: 10.1371/journal.pone.0268415 (PMC10101424; doi:10.1371/journal.pone.0268415)
Supplement: S2 Table — For each dietary condition, half of the flies were inoculated and half were treated as controls. All flies were reared on a cornmeal diet until age 12 from egg. Then the specific dietary conditions were applied. (PDF) [file pone.0268415.s003.pdf]

Table S2. Dietary treatments of Experiment 4.

For each dietary condition, half of the flies were inoculated and half were treated as controls. All flies were reared on a cornmeal diet until age 12 from egg. Then the specific dietary conditions were applied.

Treatment name on Fig. 5	Day 12 – 14 (before spray)	Day 15 – 27 (after spray)
C/C	Cornmeal	Cornmeal
C/CY	Cornmeal	Cornmeal with yeast supplement
CY/C	Cornmeal with yeast supplement	Cornmeal
CY/CY	Cornmeal with yeast supplement	Cornmeal with yeast supplement
G/G	Glucose	Glucose
