# Supplementary material for: Immune defense in Drosophila melanogaster depends on diet, sex, and mating status
Source: PLoS One. 2023 Apr 13;18(4):e0268415. doi: 10.1371/journal.pone.0268415 (PMC10101424; doi:10.1371/journal.pone.0268415)
Supplement: S4 Table — Hazard ratios and p-Values are presented in the time intervals of 0–5, 5–11, and 11–21 days post inoculation. (PDF) [file pone.0268415.s005.pdf]

Table S4. Hazard ratios and p-Values when comparing mating statuses in both control and fungal inoculated Drosophila melanogaster (Experiment 1).

Hazard ratios and p-Values are presented in the time intervals of 0-5, 5-11, and 11-21 days post inoculation.

Treatment	Sex	Hazard ratios between mating status	0 – 5	5 – 11	11 – 21
Control	Female	Mated vs Virgins *(p-value)*	1.28 *(0.4978)*	1.24 *(0.4446)*	0.93 *(0.8012)*
Control	Female	Cohabiting vs Virgins *(p-value)*	2.79 *(0.0014)*	4.59 *(<0.0001)*	2.29 *(<0.0001)*
Control	Female	Cohabiting vs Mated *(p-value)*	2.19 *(0.0219)*	3.69 *(0.0003)*	2.46 *(0.0046)*
Control	Male	Mated vs Virgins *(p-value)*	0.70 *(0.3297)*	0.69 *(0.1956)*	0.52 *(0.0238)*
Control	Male	Cohabiting vs Virgins *(p-value)*	2.79 *(0.0014)*	4.59 *(<0.0001)*	2.29 *(<0.0001)*
Control	Male	Cohabiting vs Mated *(p-value)*	3.96 *(0.0005)*	6.69 *(<0.0001)*	4.45 *(<0.0001)*
Inoculated	Female	Mated vs Virgins *(p-value)*	2.72 *(0.0041)*	2.65 *(<0.0001)*	1.99 *(<0.0001)*
Inoculated	Female	Cohabiting vs Virgins *(p-value)*	2.79 *(0.0014)*	4.59 *(<0.0001)*	2.29 *(<0.0001)*
Inoculated	Female	Cohabiting vs Mated *(p-value)*	1.03 *(0.9073)*	1.73 *(0.0001)*	1.15 *(0.3419)*
Inoculated	Male	Mated vs Virgins *(p-value)*	1.50 *(0.1854)*	1.46 *(0.0098)*	1.10 *(0.4450)*
Inoculated	Male	Cohabiting vs Virgins *(p-value)*	2.79 *(0.0014)*	4.59 *(<0.0001)*	2.29 *(<0.0001)*
Inoculated	Male	Cohabiting vs Mated *(p-value)*	1.86 *(0.0178)*	3.14 *(<0.0001)*	2.09 *(0.0002)*
