# Supplementary material for: Immune defense in Drosophila melanogaster depends on diet, sex, and mating status
Source: PLoS One. 2023 Apr 13;18(4):e0268415. doi: 10.1371/journal.pone.0268415 (PMC10101424; doi:10.1371/journal.pone.0268415)
Supplement: S5 Table — Hazard ratios and p-values are presented for days 0–21 post inoculation. (PDF) [file pone.0268415.s006.pdf]

Table S5. Hazard ratios and p-Values when comparing males and females under different mating statuses in both control and fungal inoculated Drosophila melanogaster (Experiment 1).

Hazard ratios and p-values are presented for days 0-21 post inoculation.

Treatment	Mating status	Hazard ratios between sex	0 – 21
Control	Virgins	Male vs Female *(p-value)*	2.11 *(0.0016)*
Control	Mated	Male vs Female *(p-value)*	1.16 *(0.4805)*
Control	Cohabiting	Male vs Female *(p-value)*	1.33 *(0.0013)*
Inoculated	Virgins	Male vs Female *(p-value)*	2.11 *(0.0016)*
Inoculated	Mated	Male vs Female *(p-value)*	0.74 *(0.0084)*
Inoculated	Cohabiting	Male vs Female *(p-value)*	1.33 *(0.0013)*
