# Supplementary material for: Immune defense in Drosophila melanogaster depends on diet, sex, and mating status
Source: PLoS One. 2023 Apr 13;18(4):e0268415. doi: 10.1371/journal.pone.0268415 (PMC10101424; doi:10.1371/journal.pone.0268415)
Supplement: S6 Table — Significantly smaller p-value of Treatment (<0.0001) indicates that the data strongly supports the difference on offspring counts between the fungal inoculated groups and the controls. The data also strongly supports that cohabiting and mated groups differ in their offspring counts (p-value = <0.0001). However, inoculated cohabiting and inoculated mated groups did not show a significant difference in offspring counts (see Fig 2 description, p-value: 0.1771). * p-value ≤ 0.05, *** p-value ≤ 0.001. (PDF) [file pone.0268415.s007.pdf]

Table S6. Table representing the interaction between days and treatment between the Control and Fungal inoculated reproductive output of the flies (Experiment 1).
Significantly smaller p-value of Treatment (<0.0001) indicates that the data strongly supports the difference on offspring counts between the fungal inoculated groups and the controls. The data also strongly supports that cohabiting and mated groups differ in their offspring counts (p-value = <0.0001). However, inoculated cohabiting and inoculated mated groups did not show a significant difference in offspring counts (see Figure 2 description, p-value: 0.1771).
* p-value ≤ 0.05, *** p-value ≤ 0.001

	Df	Sum Sq	Mean Sq	F value	Pr(>F)
Day	1	55.19	55.19	5.8532	0.0163*
Treatment	1	363.70	363.70	38.5723	<0.0001***
Mating_status	1	229.53	229.53	24.3426	<0.0001***
Treatment:Mating_status	1	47.40	47.40	5.0270	0.0259*
Day:Treatment	1	163.88	163.88	17.3799	<0.0001***
Residuals	226	2130.98	9.43		
