# Supplementary material for: Immune defense in Drosophila melanogaster depends on diet, sex, and mating status
Source: PLoS One. 2023 Apr 13;18(4):e0268415. doi: 10.1371/journal.pone.0268415 (PMC10101424; doi:10.1371/journal.pone.0268415)
Supplement: S8 Table — The hazard ratios indicate the risk of male flies dying post inoculation in comparison to the female flies. The data strongly supports that there is sexual dimorphism in post inoculation survival in the 0–8 and 8–12 age intervals. However the p-value in the 12–21 days interval suggests there is no sexual dimorphism in survival in this interval. (PDF) [file pone.0268415.s009.pdf]

Table S8. Hazard ratios and p-values for males vs females in GHA inoculated flies (Experiment 2).

The hazard ratios indicate the risk of male flies dying post inoculation in comparison to the female flies. The data strongly supports that there is sexual dimorphism in post inoculation survival in the 0-8 and 8-12 age intervals. However the p-value in the 12-21 days interval suggests there is no sexual dimorphism in survival in this interval.

Treatment	Hazard ratios between sex	0 – 8	8 – 12	12 – 21
GHA	Male vs Female<br>*(p-value)*	0.403<br>*(0.0004)*	1.268<br>*(0.0349)*	1.245<br>*(0.0749)*
