# Supplementary material for: Immune defense in Drosophila melanogaster depends on diet, sex, and mating status
Source: PLoS One. 2023 Apr 13;18(4):e0268415. doi: 10.1371/journal.pone.0268415 (PMC10101424; doi:10.1371/journal.pone.0268415)
Supplement: S10 Table — There was no sexual dimorphism in survival among the control treatments, regardless of diet. However, among fungal inoculated flies, males survived better than females in some age intervals on every diet. The timing of introduction of a glucose diet has an impact on the Hazard ratio among inoculated flies. Statistically significant differences are in bold. (PDF) [file pone.0268415.s011.pdf]

Table S10. Diet affects sexual dimorphism of *D. melanogaster* inoculated with *B. bassiana* strain GHA in a sex-specific manner (Experiment 3).

There was no sexual dimorphism in survival among the control treatments, regardless of diet. However, among fungal inoculated flies, males survived better than females in some age intervals on every diet. The timing of introduction of a glucose diet has an impact on the Hazard ratio among inoculated flies.

Statistically significant differences are in Bold.

Treatment	Diet	Hazard ratios between Sex	0 – 3	3 – 5	5 – 8	8 – 9
Control	C/C	Female vs Male *(p-value)*	0.825 *(0.5366)*	0.249 *(0.2202)*	0.664 *(0.6559)*	0.398 *(0.2762)*
Control	C/G	Female vs Male *(p-value)*	0.825 *(0.5366)*	0.249 *(0.2202)*	0.664 *(0.6559)*	0.398 *(0.2762)*
Control	G/C	Female vs Male *(p-value)*	0.825 *(0.5366)*	0.249 *(0.2202)*	0.664 *(0.6559)*	0.398 *(0.2762)*
Control	G/G	Female vs Male *(p-value)*	0.825 *(0.5366)*	0.249 *(0.2202)*	0.664 *(0.6559)*	0.398 *(0.2762)*
Inoculated	C/C	Female vs Male *(p-value)*	1.371 *(0.4307)*	2.780 *(0.0863)*	**5.377** ***(<0.0001)***	1.203 *(0.4769)*
Inoculated	C/G	Female vs Male *(p-value)*	5.385e+06 *(0.9925)*	1.505 *(0.5299)*	**10.071** ***(<0.0001)***	**6.116** ***(0.0016)***
Inoculated	G/C	Female vs Male *(p-value)*	1.500 *(0.5326)*	4.537 *(0.0589)*	**6.252** ***(<0.0001)***	1.443 *(0.1193)*
Inoculated	G/G	Female vs Male *(p-value)*	0.249 *(0.0848)*	1.486 *(0.6661)*	**33.574** ***(0.0011)***	**8.394** ***(0.0002)***
