# Supplementary material for: Immune defense in Drosophila melanogaster depends on diet, sex, and mating status
Source: PLoS One. 2023 Apr 13;18(4):e0268415. doi: 10.1371/journal.pone.0268415 (PMC10101424; doi:10.1371/journal.pone.0268415)
Supplement: S11 Table — Effects of inoculation and diet changes on males and females compared to control males on cornmeal diet. (PDF) [file pone.0268415.s012.pdf]

Table S11. Effects of dietary condition and infection status on male and female survival in different age groups (Experiment 3).
Effects of inoculation and diet changes on males and females compared to control males on cornmeal diet.

Sex	Treatment	Hazard ratios between Diet	0 – 3	3 – 5	5 – 8	8 – 9
Male	Control	C/C *(baseline)*	1	1	1	1
Male	Control	C/G *(p-value)*	1.880 *(0.1978)*	1.005 *(0.9999)*	1.199 *(0.8982)*	0.943 *(0.9902)*
Male	Control	G/C *(p-value)*	0.451 *(0.2543)*	1.487e+07 *(0.9940)*	1.054 *(0.9940)*	4.228 *(0.2034)*
Male	Control	G/G *(p-value)*	**2.970** ***(0.0161)***	9.393e+06 *(0.9941)*	1.995 *(0.5755)*	2.001 *(0.5739)*
Male	Inoculated	C/C *(p-value)*	3.059 *(0.9902)*	2.534e+07 *(0.9938)*	**53.097** ***(0.0005)***	**50.792** ***(0.0005)***
Male	Inoculated	C/G *(p-value)*	1.025e-07 *(0.9922)*	2.491e+07 *(0.9938)*	8.866 *(0.0647)*	6.001 *(0.1235)*
Male	Inoculated	G/C *(p-value)*	1.037 *(0.9548)*	1.173e+07 *(0.9940)*	**49.200** ***(0.0007)***	**54.400** ***(0.0004)***
Male	Inoculated	G/G *(p-value)*	2.046 *(0.1881)*	1.158e+07 *(0.9940)*	1.643 *(0.7355)*	5.539 *(0.1407)*
Female	Control	C/C *(p-value)*	1	1	1	1
Female	Control	C/G *(p-value)*	1.880 *(0.1978)*	1.005 *(0.9999)*	1.199 *(0.8982)*	0.943 *(0.9902)*
Female	Control	G/C *(p-value)*	0.451 *(0.2543)*	1.487e+07 *(0.9940)*	1.054 *(0.9940)*	4.228 *(0.2034)*
Female	Control	G/G *(p-value)*	**2.970** ***(0.0161)***	9.393e+06 *(0.9941)*	1.995 *(0.5755)*	2.001 *(0.5739)*
Female	Inoculated	C/C *(p-value)*	**5.086** ***(0.0017)***	2.824e+08 *(0.9931)*	**4.299e+02** ***(<0.0001)***	**1.536e+02** ***(<0.0001)***
Female	Inoculated	C/G *(p-value)*	0.669 *(0.6265)*	1.503e+08 *(0.9931)*	**1.344e+02** ***(0.0001)***	**1.266e+02** ***(0.0002)***
Female	Inoculated	G/C *(p-value)*	1.888 *(0.2802)*	2.133e+08 *(0.9930)*	**4.632e+02** ***(<0.0001)***	**1.973e+02** ***(<0.0001)***

Female	Inoculated	G/G<br>*(p-value)*	0.617<br>*(0.5587)*	6.902e+07<br>*(0.9934)*	**83.040**<br>***(0.0004)***	**85.140**<br>***(0.0005)***
