# Supplementary material for: Immune defense in Drosophila melanogaster depends on diet, sex, and mating status
Source: PLoS One. 2023 Apr 13;18(4):e0268415. doi: 10.1371/journal.pone.0268415 (PMC10101424; doi:10.1371/journal.pone.0268415)
Supplement: S13 Table — Hazard ratios and p-values are presented from 0–4, 4–9 and 9–14 days post inoculation. (PDF) [file pone.0268415.s014.pdf]

Table S13. Effect of diet on survival of males and females combined when inoculated with B. bassiana GHA (Experiment 4).

Hazard ratios and p-values are presented from 0-4, 4-9 and 9-14 days post inoculation.

Treatment	Sex	Hazard ratios between Diet	0 – 4	4 – 9	9 – 12
Control	Male	C/CY vs C/C *(p-value)*	1.443 *(0.5725)*	0.383 *(0.2577)*	0.190 *(0.1370)*
Control	Male	CY/C vs C/C *(p-value)*	1.188 *(0.7990)*	1.331 *(0.6275)*	1.144 *(0.8257)*
Control	Male	CY/CY vs C/C *(p-value)*	3.158 *(0.0551)*	2.338 *(0.1281)*	0.946 *(0.9344)*
Control	Male	G/G vs C/C *(p-value)*	1.203 *(0.7846)*	1.542 *(0.4511)*	8.814e-07 *(0.9763)*
Control	Female	C/CY vs C/C *(p-value)*	1	1	1
Control	Female	CY/C vs C/C *(p-value)*	1	1	1
Control	Female	CY/CY vs C/C *(p-value)*	1	1	1
Control	Female	G/G vs C/C *(p-value)*	1	1	1
Inoculated	Male	C/CY vs C/C *(p-value)*	0.577 *(0.3288)*	**0.218** ***(<0.0001)***	**0.334** ***(<0.0001)***
Inoculated	Male	CY/C vs C/C *(p-value)*	0.453 *(0.1946)*	**0.553** ***(0.0001)***	**0.497** ***(<0.0001)***
Inoculated	Male	CY/CY vs C/C *(p-value)*	0.644 *(0.4090)*	**0.216** ***(<0.0001)***	**0.221** ***(<0.0001)***
Inoculated	Male	G/G vs C/C *(p-value)*	0.813 *(0.6830)*	**0.059** ***(<0.0001)***	**0.041** ***(<0.0001)***
Inoculated	Female	C/CY vs C/C *(p-value)*	0.628 *(0.3339)*	**0.282** ***(<0.0001)***	**0.284** ***(<0.0001)***

Inoculated	Female	CY/C vs C/C<br>*(p-value)*	0.535<br>(0.2171)	**0.467**<br>***(<0.0001)***	**0.479**<br>***(<0.0001)***
Inoculated	Female	CY/CY vs C/C<br>*(p-value)*	0.462<br>*(0.1540)*	**0.155**<br>***(<0.0001)***	**0.261**<br>***(<0.0001)***
Inoculated	Female	G/G vs C/C<br>*(p-value)*	0.7981<br>*(0.6115)*	**0.348**<br>***(<0.0001)***	**0.222**<br>***(<0.0001)***
