# Supplementary material for: Immune defense in Drosophila melanogaster depends on diet, sex, and mating status
Source: PLoS One. 2023 Apr 13;18(4):e0268415. doi: 10.1371/journal.pone.0268415 (PMC10101424; doi:10.1371/journal.pone.0268415)
Supplement: S14 Table — Whenever yeast supplementation was provided after inoculation, it reduced the sexual dimorphism in survival. When yeast supplementation was provided before and after the spray, there was sexual dimorphism among uninfected flies. The hazard ratio, showing difference in hazard between males and females is largest for flies that received the glucose diet. (PDF) [file pone.0268415.s015.pdf]

Table S14. Yeast supplementation affects sexual dimorphism in surviving infection (Experiment 4).

Whenever yeast supplementation was provided after inoculation, it reduced the sexual dimorphism in survival. When yeast supplementation was provided before and after the spray, there was sexual dimorphism among uninfected flies. The hazard ratio, showing difference in hazard between males and females is largest for flies that received the glucose diet.

Treatment	Diet	Hazard ratios between Sex	0 – 4	4 – 9	9 – 12
Control	C/C	Female vs Male *(p-value)*	1.300 *(0.2228)*	0.880 *(0.7970)*	0.680 *(0.4515)*
Control	C/CY	Female vs Male *(p-value)*	0.901 *(0.8189)*	2.296 *(0.2665)*	3.569 *(0.2228)*
Control	CY/C	Female vs Male *(p-value)*	1.09 *(0.8538)*	0.661 *(0.3448)*	0.594 *(0.9770)*
Control	CY/CY	Female vs Male *(p-value)*	**0.412** ***(0.0175)***	**0.376** ***(0.0138)***	0.718 *(0.5546)*
Control	G/G	Female vs Male *(p-value)*	1.081 *(0.8734)*	0.570 *(0.1805)*	7.709e+05 *(0.9700)*
Inoculated	C/C	Female vs Male *(p-value)*	1.215 *(0.6606)*	**1.832** ***(<0.0001)***	0.950 *(0.6735)*
Inoculated	C/CY	Female vs Male *(p-value)*	1.324 *(0.2228)*	2.364 *(0.2228)*	0.806 *(0.1728)*
Inoculated	CY/C	Female vs Male *(p-value)*	1.434 *(0.5796)*	**1.549** ***(0.0040)***	0.915 *(0.4867)*
Inoculated	CY/CY	Female vs Male *(p-value)*	0.871 *(0.6606)*	1.312 *(0.2391)*	1.123 *(0.4867)*
Inoculated	G/G	Female vs Male *(p-value)*	1.193 *(0.7279)*	**10.748** ***(<0.0001)***	**5.126** ***(<0.0001)***
