# Supplementary material for: Immune defense in Drosophila melanogaster depends on diet, sex, and mating status
Source: PLoS One. 2023 Apr 13;18(4):e0268415. doi: 10.1371/journal.pone.0268415 (PMC10101424; doi:10.1371/journal.pone.0268415)
Supplement: S16 Table — Pairwise comparisons of survival are shown, comparing the control (no yeast) condition to the lowest yeast level, comparing the lowest and intermediate yeast levels to each other, and comparing the intermediate and high yeast levels to each other. In both males and females, survival is improved by low and intermediate levels of yeast supplementation. (PDF) [file pone.0268415.s017.pdf]

Table S16. Intermediate levels of yeast supplementation improved survival (Experiment 5). Pairwise comparisons of survival are shown, comparing the control (no yeast) condition to the lowest yeast level, comparing the lowest and intermediate yeast levels to each other, and comparing the intermediate and high yeast levels to each other. In both males and females, survival is improved by low and intermediate levels of yeast supplementation.

Treatment	Sex	Hazard ratios between Diet	0 – 5	5 – 8	8 – 12
Inoculated	Male	CY0.5 vs C *(p-value)*	1.303 *(0.3577)*	**0.283** ***(0.0243)***	**0.508** ***(0.0113)***
Inoculated	Male	CY1.0 vs CY0.5 *(p-value)*	0.931 *(0.7229)*	1.002 *(0.9941)*	**0.676** ***(0.0009)***
Inoculated	Male	CY1.5 vs CY1.0 *(p-value)*	0.899 *(0.5556)*	1.290 *(0.0768)*	1.268 *(0.0768)*
Inoculated	Female	CY0.5 vs C *(p-value)*	**0.537** ***(0.0243)***	**0.496** ***(<0.0001)***	**0.749** ***(0.0113)***
Inoculated	Female	CY1.0 vs CY0.5 *(p-value)*	0.883 *(0.6568)*	**0.464** ***(<0.0001)***	**0.644** ***(0.0004)***
Inoculated	Female	CY1.5 vs CY1.0 *(p-value)*	0.899 *(0.5556)*	1.290 *(0.0768)*	1.268 *(0.0768)*
