# Supplementary material for: Immune defense in Drosophila melanogaster depends on diet, sex, and mating status
Source: PLoS One. 2023 Apr 13;18(4):e0268415. doi: 10.1371/journal.pone.0268415 (PMC10101424; doi:10.1371/journal.pone.0268415)
Supplement: S17 Table — When flies were inoculated with fungus, there was sexual dimorphism on all diets, but the age intervals and magnitudes of this dimorphism changed with the level of yeast supplementation. When there is no yeast supplement or a little amount of supplement, the dimorphism starts at earlier ages than with higher levels of yeast supplement. (PDF) [file pone.0268415.s018.pdf]

Table S17. There was no sexual dimorphism in survival among control flies (Experiment 5).

When flies were inoculated with fungus, there was sexual dimorphism on all diets, but the age intervals and magnitudes of this dimorphism changed with the level of yeast supplementation. When there is no yeast supplement or a little amount of supplement, the dimorphism starts at earlier ages than with higher levels of yeast supplement.

Treatment	Diet	Hazard ratios between Sex	0 – 5	5 – 8	8 – 12
Control	C	Female vs Male *(p-value)*	1.065 *(0.7612)*	1.145 *(0.6556)*	1.030 *(0.9010)*
Control	CY0.5	Female vs Male *(p-value)*	1.065 *(0.7612)*	1.145 *(0.6556)*	1.030 *(0.9010)*
Control	CY1.0	Female vs Male *(p-value)*	1.065 *(0.7612)*	1.145 *(0.6556)*	1.030 *(0.9010)*
Control	CY1.5	Female vs Male *(p-value)*	1.065 *(0.7612)*	1.145 *(0.6556)*	1.030 *(0.9010)*
Inoculated	C	Female vs Male *(p-value)*	**1.711** ***(0.0261)***	**4.794** ***(<0.0001)***	0.854 *(0.1147)*
Inoculated	CY0.5	Female vs Male *(p-value)*	**0.936** ***(0.0261)***	**4.404** ***(<0.0001)***	1.192 *(0.1002)*
Inoculated	CY1.0	Female vs Male *(p-value)*	0.938 *(0.7534)*	**2.805** ***(<0.0001)***	1.043 *(0.6100)*
Inoculated	CY1.5	Female vs Male *(p-value)*	0.938 *(0.7534)*	**2.805** ***(<0.0001)***	1.043 *(0.6100)*
